# The Pragmatic Approach or Simply the Better Approach?

**DOI:** 10.1016/j.jacadv.2025.102138

**Published:** 2025-09-09

**Authors:** Ravi Agarwala

**Affiliations:** LeBauer Brodie Center for Cardiovascular Research and Education, Cone Health, Greensboro, North Carolina, USA

Khalid et al^1^ thoughtfully discuss the issue of cardiac intensive care staffing advocating for a pragmatic, multidisciplinary approach to physician staffing in the cardiac intensive care unit.

Although I agree with the general thrust of the paper, as a former assistant program director, I feel that it sells critical care medicine (CCM) physicians short. Rare are the CCM programs that do not teach airway support. All our fellows were comfortable with pulmonary artery catheter placement, and cardiac postoperative care was a prominent portion of our program, and it is included in many other CCM programs as are extra-corporeal membrane oxygenation and other forms of mechanical cardiac support. Furthermore, the realities of community practice, which nowadays includes large tertiary care programs, often require a scope of practice broader than those defined by academic training programs. Indeed, long before the concept of cardiology critical care blossomed into existence, CCM physicians had working as de facto cardiac intensivists.

Many of them were self-taught, and this, I believe highlights a further point of friction in the development of the field, namely the increasing reliance on formal fellowship training for every skill. The reality is that no training program perfectly prepares a physician for practice, especially as the expectations will vary based on the different critical care ecosystems throughout the country. Critical care training in the United Sates is not as standardized as it in other countries, such as Canada or Australasia, where there is one training standard across all base specialties. Cardiac critical care illustrates the difficulty of a training system that promotes formal fellowship training as the exclusive route to acquire increasingly narrower specialization as such an approach lacks the flexibility to respond to rapid advances medical care.

Thus, the pragmatic approach proposed could be strengthened by acknowledging the need for “on-the-job” training, and organizations like the American College of Cardiology could contribute to this new educational paradigm by sponsoring the coursework and certifications that cover different aspects of cardiovascular critical care for practicing physicians, modeled on the program currently offered for advanced practice providers, open to all who are involved in cardiovascular critical care. Finally, one of the great strengths and joys of working in a multidisciplinary team is the opportunity to learn from colleagues every day. Even the gold-standard critical care cardiologist will not have the breath of critical care experience as the general intensivist or the airway skills of the anesthesiologist. We are taught that there is strength in diversity, and why should this not be the case in the cardiac intensive care unit? Perhaps the pragmatic approach is, in fact, the best approach.

## References

[1] Khalid S., Ma J., Janjua S., Taub C.C., Maxey-Jones C.L. (2025). But we do not have a critical care cardiologist? A pragmatic approach to staffing the cardiac intensive care unit. JACC Adv.

